# Activation of mucosal-associated invariant T cells in the lungs of sarcoidosis patients

**DOI:** 10.1038/s41598-019-49903-6

**Published:** 2019-09-12

**Authors:** Hisayo Matsuyama, Takuma Isshiki, Asako Chiba, Tetsuo Yamaguchi, Goh Murayama, Yoshikiyo Akasaka, Yoshinobu Eishi, Susumu Sakamoto, Sakae Homma, Sachiko Miyake

**Affiliations:** 1 Department of Respiratory Medicine, Toho University Graduate School of Medicine, Tokyo, Japan; 2 Department of Immunology, Juntendo University School of Medicine, Tokyo, Japan; 3 Shinjuku Tsurukame Clinic, Tokyo, Japan; 4 Department of Internal Medicine and Rheumatology, Juntendo University School of Medicine, Tokyo, Japan; 5 Department of Pathology, Toho University Omori Medical Center, Tokyo, Japan; 6 Department of human Pathology, Tokyo Medical and Dental University, Tokyo, Japan

**Keywords:** Innate lymphoid cells, Predictive markers

## Abstract

Although the pathogenesis of sarcoidosis is not fully understood, immunological characterization has elucidated highly polarized expression of the type 1 T helper cell response. Mucosal-associated invariant T (MAIT) cells are innate T cells that recognize bacterial riboflavin and rapidly produce cytokines such as interferon γ and tumor necrosis factor α. We prospectively evaluated the proportion of MAIT cells and the expression levels of cell surface markers in peripheral blood from 40 sarcoidosis patients and 28 healthy controls. MAIT cells in bronchoalveolar lavage fluid (BALF) were also examined in 12 sarcoidosis patients. In peripheral blood, the proportion of MAIT cells was lower (P = 0.0002), but the expression levels of CD69 and programmed death 1 on MAIT cells were higher in sarcoidosis patients than in healthy controls. Moreover, CD69 expression levels were significantly correlated with clinical biomarkers. Sarcoidosis patients with parenchymal infiltration in the lungs showed a significantly higher proportion and number of MAIT cells in BALF compared to patients without parenchymal infiltration. CD69 expression levels on MAIT cells in BALF were higher than levels in peripheral blood. The activation status of MAIT cells might reflect the disease activity of sarcoidosis. Therefore, it is a potential target for sarcoidosis treatment.

## Introduction

Sarcoidosis is a systemic inflammatory disorder that is histologically characterized by noncaseating epithelioid granulomas^1^. Previous studies have reported that various types of immune cells and cytokines orchestrate the immune response that leads to granuloma formation in multiple organs. Th1-type immunity has been implicated in the immune response of sarcoidosis^2–6^. Interferon γ (IFN-γ) levels are elevated in bronchoalveolar lavage fluid (BALF) from sarcoidosis patients^2^, and accumulation of Th1 CD4 T cells is observed in granuloma lesions^3^. In addition, concentrations of cytokines such as interleukin (IL)-12, IL-18, and IL-27, which promote the Th1 response, are increased in sarcoidosis tissues^2,4,5^. Moreover, tumor necrosis factor α (TNF-α) is important in granuloma formation. Macrophages isolated from liver granulomas produce TNF-α, and TNF-α anti-serum shrinks liver granulomas in mice^6^. In human sarcoidosis, TNF-α production in alveolar macrophages is higher in sarcoidosis patients than in healthy controls and reflects disease activity^7,8^.

Innate T cells are a T cell subset that is distinct from conventional T cells and express semi-invariant T cell receptors (TCRs). They are present in mucosal and epithelial tissues and rapidly exert effector functions against exogenous stimuli, without clonal expansion. Interest is growing in the roles of these cells in various types of immune responses. Two subtypes of innate T cells have been reported: invariant natural killer T (iNKT) cells and mucosal-associated invariant T (MAIT) cells. iNKT cells express the invariant TCR α and are restricted by the major histocompatibility complex molecule CD1d^9,10^. Several studies have shown that iNKT cells are involved in sarcoidosis pathogenesis^11,12^. In these studies, the number of iNKT cells in peripheral blood is lower in sarcoidosis patients, and indeed, these cells are functionally exhausted in these patients. The proportion of MAIT cells in human peripheral blood is 1–10% of CD3^+^ cells^13–15^, which is 10- to 1000-fold as abundant as iNKT cells. Therefore, MAIT cells may play an important role in the human immune response^16^.

MAIT cells also express an invariant TCR α-chain and are restricted by the nonpolymorphic major histocompatibility complex class 1b molecule MR1^17–19^. Bacteria-derived vitamin B2 metabolites bind to MR1 and activate MAIT cells^20^. Previous studies have reported that MAIT cells are activated in an MR1-dependent manner by various bacterial species, including *Mycobacterium tuberculosis*, *Escherichia coli*, and *Shigella flexneri*^21–24^. Thus, MAIT cells are thought to be associated with antimicrobial immunity. In addition, these cells are implicated in various types of immune responses such as autoimmune diseases. Functionally, MAIT cells can produce a large number of cytokines, such as IFN-γ, TNF-α, and IL-17, following stimulation by microbial antigens and cytokines^13,14,21,25,26^. In addition, previous studies have noted a critical role for MAIT cells in another lung granulomatous disease, *Mycobacterium tuberculosis* infection^21,22,27^. We thus hypothesized that MAIT cells contribute to sarcoidosis pathogenesis and that activation of MAIT cells reflects sarcoidosis disease activity. To test these hypotheses, we analyzed MAIT cells in peripheral blood and BALF of sarcoidosis patients.

## Results

### MAIT cells reacted to stimulation by *C. acnes in vitro*

**Figure 1:**
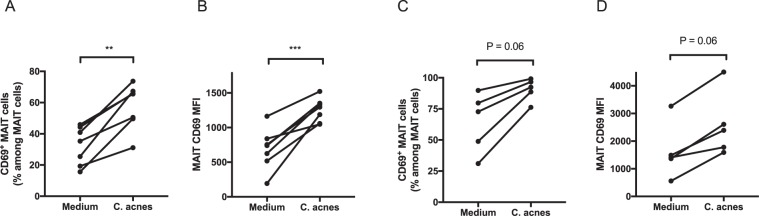
Mucosal-associated invariant T (MAIT) cells were activated by *Cutibacterium acnes* (*C. acnes)* stimulation *in vitro*. The relative levels of CD69^+^ MAIT cells were higher when MAIT cells were stimulated with *C. acnes* than in the absence of such stimulation. The proportion of CD69^+^ cells and mean fluorescence intensity (MFI) of CD69 on MAIT cells were determined in healthy controls (**A** and **B**, respectively; n = 7) and sarcoidosis patients (**C** and **D**, respectively; n = 5). The baseline characteristics of healthy controls (n = 7) and sarcoidosis patients (n = 5) are shown in Supplementary Table S1. The black circles represent individual participants. *P < 0.05, **P < 0.01, ***P < 0.001.

*Cutibacterium acnes* (*C. acnes*) is a probable causative microorganism of sarcoidosis^28–32^. In addition, *C. acnes* utilizes the riboflavin metabolism pathway according to the Kyoto Encyclopedia of Genes and Genomes database^33^. Thus, we first examined if MAIT cells responded to stimulation by *C. acnes* by testing this possibility in peripheral blood mononuclear cells (PBMCs) from healthy controls. After stimulation by *C. acnes* and CD28, the expression level of the activation marker CD69 and the proportion of CD69^+^ MAIT cells among all MAIT cells were higher than in unstimulated control cells (P = 0.005 and P = 0.0009, respectively) (Fig. 1A-B). These results indicate that MAIT cells in peripheral blood can respond to *C. acnes*. Next, we examined the activation of MAIT cells from sarcoidosis patients following *C. acnes* stimulation. MAIT cells from sarcoidosis patients also showed greater CD69 expression after *C. acnes* stimulation; however, the proportion of CD69^+^ MAIT cells did not significantly differ between unstimulated cells and *C. acnes*-stimulated cells (P = 0.05 and P = 0.20, respectively) (Fig. 1C-D), probably because MAIT cells in unstimulated PBMCs already highly express CD69.

### Patient characteristics, proportions of MAIT cells, and cell surface markers on MAIT cells in peripheral blood from sarcoidosis patients

We found that MAIT cells were activated by the probable causative microorganism of sarcoidosis. Therefore, we next investigated the proportion and cell surface markers on MAIT cells in peripheral blood and BALF from sarcoidosis patients. The baseline characteristics of the 40 patients with sarcoidosis and 28 healthy age- and sex-matched controls are summarized in Table 1. Approximately 30% of patients had been treated with immunosuppressants such as corticosteroids and methotrexate; the remaining patients had not received medication for sarcoidosis. BALF was obtained from 14 patients for the purpose of diagnosing sarcoidosis. The findings revealed a high CD4/8 ratio and elevated lymphocyte differential count, which are findings consistent with sarcoidosis.

**Table 1:** Characteristics of sarcoidosis patients and healthy controls.

	HC (n = 28)	SA (n = 40)
Age (years)	54 ± 11	57 ± 12
Male, n (%)	12 (40)	19 (44)
Smoking history (never/former/current)	23/2/3	17/11/7
Duration of disease (years)		10 ± 11
Number of involved organs (1/2/3/4/5)		7/14/13/5/1
Stage of pulmonary lesions (0/1/2/3/4)		6/14/9/4/7
Ongoing treatment (none/CS/MTX)		19/14/7
Pathologically confirmed disease, n (%)		33 (83)
BALF findings (n=14)		
CD4/8		4 ± 3
Lymphoid cells (%)		17 ± 12
Macrophages (%)		80 ± 12

n (%) or mean ± standard deviation.

Abbreviations: HC, healthy controls; SA, patients with sarcoidosis; CS, corticosteroids; MTX, methotrexate; BALF, bronchoalveolar lavage fluid.

**Figure 2:**
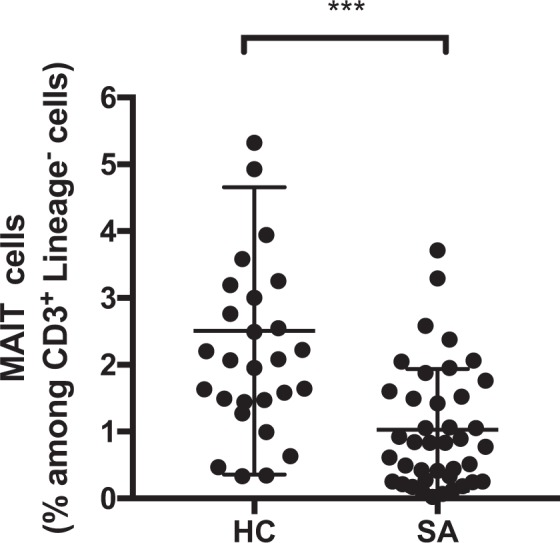
Frequency of mucosal-associated invariant T (MAIT) cells in peripheral blood of sarcoidosis patients (SA) (n = 40) and healthy controls (HC) (n = 28). The proportion of MAIT cells in peripheral blood was lower in SA than in HC (1.03 ± 0.14% vs. 2.51 ± 0.40%, respectively; P = 0.0002). The black circles represent individual participants. ***P < 0.001. Lineage^−^ is defined as CD1a^−^, CD11c^−^, CD14^−^, CD19^−^, CD34^−^, CD123^−^, CD303^−^, T cell receptor γ/δ^−^, and FcεR1^α−^.

**Figure 3:**
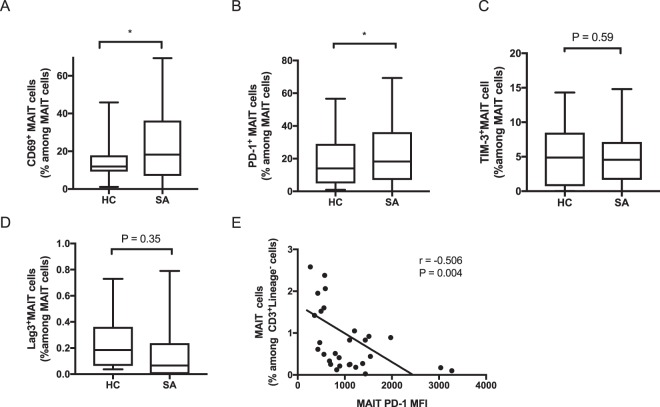
Cell surface markers on mucosal-associated invariant T (MAIT) cells in peripheral blood mononuclear cells. Flow cytometry was used to determine the proportions of CD69^+^ cells (**A**), programmed death 1 (PD-1)^+^ cells (**B**), T-cell immunoglobulin and mucin domain 3 (TIM-3)^+^ cells (**C**), and lymphocyte activation gene-3 (LAG-3)^+^ cells (**D**) among total MAIT cells in healthy controls (HC) (n = 28) and sarcoidosis patients (SA) (n = 40). Proportions of CD69^+^ cells and PD-1^+^ cells were significantly higher in SA. (**E**) Correlation of mean fluorescence intensity (MFI) of PD-1 on MAIT cells with the proportion of MAIT cells among Lineage^−^ (CD1a^−^, CD11c^−^, CD14^−^, CD19^−^, CD34^−^, CD123^−^, CD303^−^, T cell receptor γ/δ^−^, and FcεR1α^−^) T cells from SA. PD-1 MFI of MAIT cells was inversely correlated with the proportion of MAIT cells (r = −0.506, P = 0.004). *P < 0.05.

We examined the proportion of MAIT cells and iNKT cells in peripheral blood from sarcoidosis patients and healthy controls (Fig. 2, Table 2). The proportion of MAIT cells was lower in sarcoidosis patients than in healthy controls (1.03 ± 0.14% vs. 2.51 ± 0.40%, respectively; P = 0.0002). In contrast, no significant difference in the proportion of iNKT cells was found between patients with sarcoidosis and healthy controls (0.053 ± 0.030% vs. 0.043 ± 0.008%, P = 0.8). The expression of cell surface markers on MAIT cells is shown in Fig. 3. The proportions of CD69+ MAIT cells and PD-1^+^ MAIT cells among all MAIT cells were significantly higher in sarcoidosis patients (P = 0.01 and P = 0.02, respectively). However, there were no significant differences in the expression levels of T-cell immunoglobulin and mucin domain 3 (TIM-3) and lymphocyte activation gene-3 (LAG-3) on MAIT cells between sarcoidosis patients and healthy controls. Interestingly, PD-1 expression on MAIT cells was negatively correlated with the proportion of MAIT cells in peripheral blood (r = −0.506, P = 0.004).

**Table 2:** Proportions of innate T cells in peripheral blood of sarcoidosis patients and healthy controls.

Innate T cells	HC (n = 28)	SA (n = 40)	P value
MAIT cells (%)	2.51 ± 0.40	1.03 ± 0.14	0.0002
iNKT cells (%)	0.043 ± 0.008	0.053 ± 0.030	N.S.

Values are expressed as mean ± standard deviation.

HC, healthy controls; SA, patients with sarcoidosis; MAIT cells, mucosal-associated invariant T cells; iNKT cells, invariant natural killer T cells. The proportions of MAIT cells and iNKT cells are those among CD3^+^ Lineage^−^ (CD1a^−^, CD11c^−^, CD14^−^, CD19^−^, CD34^−^, CD123^−^, CD303^−^, T cell receptor γ/δ^−^, and FcεR1α^−^) cells.

### Correlations with clinical variables

**Figure 4:**
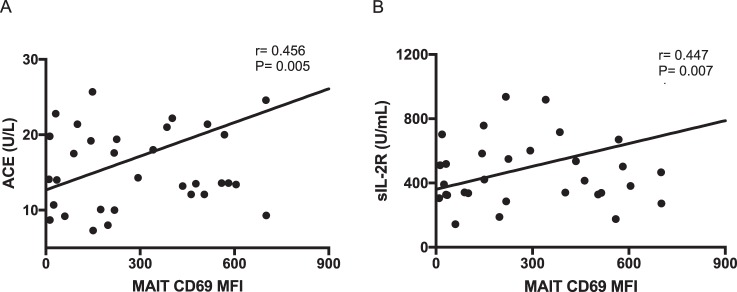
Correlations of CD69 mean fluorescence intensity (MFI) of mucosal-associated invariant T (MAIT) cells with clinical biomarkers. MFI of CD69 was significantly correlated with angiotensin-converting enzyme (ACE) (r = 0.456, P = 0.005) and soluble interleukin-2 receptor (sIL-2R) levels (r = 0.447, P = 0.007).

To clarify the associations between MAIT cell activity and sarcoidosis disease activity, we analyzed correlations between the expression levels of CD69 on MAIT cells and clinical variables. The proportion of CD69^+^ MAIT cells among all MAIT cells did not significantly differ between patients who did and did not receive corticosteroid treatment (data not shown). Similarly, the number of organs involved and chest radiographic stage were not correlated with CD69 expression on MAIT cells (data not shown). Serum angiotensin-converting enzyme (ACE) and soluble interleukin-2 receptor (sIL-2R) are useful for evaluating sarcoidosis disease activity^34–37^. Therefore, we analyzed the correlations of these clinical biomarkers with CD69 expression on MAIT cells (Fig. 4). The expression level of the activation marker CD69 on MAIT cells was significantly correlated with ACE (r = 0.456, P = 0.0005) and sIL-2R (r = 0.447, P = 0.007), which suggests that the activity of MAIT cells reflects disease activity.

### IL-18 was correlated with CD69 expression on MAIT cells from sarcoidosis patients

**Figure 5:**
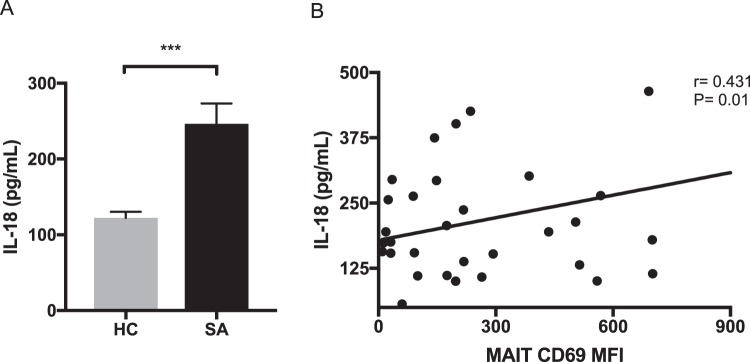
Serum interleukin (IL)-18 levels in healthy controls (HC) and sarcoidosis patients (SA). (**A**) Serum IL-18 production was higher in SA than in HC (247 ± 27 pg/mL vs. 122 ± 8 pg/mL, P = 0.0001). (**B**) The IL-18 concentration was correlated with mean fluorescence intensity (MFI) of CD69 expression on mucosal-associated invariant T (MAIT) cells in SA (r = 0.431, P = 0.01). ***P < 0.001.

Next, we measured serum levels of cytokines such as IFN-γ, TNF-α, and IL-17 to examine the association of MAIT cells with cytokines in sarcoidosis patients. However, these cytokine levels were below the detection limit by ELISA (data not shown). MAIT cells highly express IL-18 receptor α (IL-18Rα) on their surfaces and can be activated by IL-18 stimulation^38–40^. Moreover, our group and others previously revealed that IL-18 is a significant cytokine that strongly correlates with the activity of MAIT cells in other systemic inflammatory diseases^38,41,42^. Thus, we analyzed serum IL-18 levels in sarcoidosis patients and healthy controls (Fig. 5), and found that they were significantly higher in sarcoidosis patients than in healthy controls (247 ± 27 pg/mL vs. 122 ± 8 pg/mL, P = 0.0001; Fig. 5A). In addition, the level of CD69 expression on MAIT cells was positively correlated with the IL-18 concentration in the serum of sarcoidosis patients (r = 0.431, P = 0.01; Fig. 5B). These findings suggest that IL-18 is an activator of MAIT cells in sarcoidosis patients.

### Activation of MAIT cells in the lungs of sarcoidosis patients

**Figure 6:**
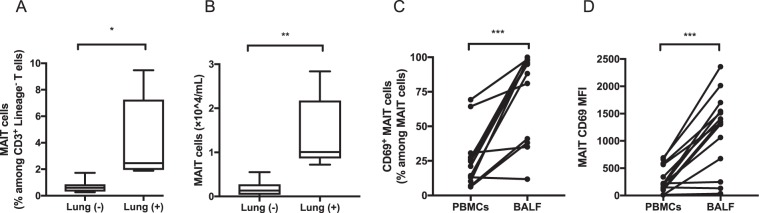
(**A**,**B**) Proportion and frequency of mucosal-associated invariant T (MAIT) cells in bronchoalveolar lavage fluid (BALF) from sarcoidosis patients (n = 14). The absolute number (**A**) and proportion (**B**) of MAIT cells were higher in BALF from lungs of patients with parenchymal infiltration (Lung (+)) than in lungs of those without parenchymal infiltration (Lung (−)). The proportion of CD69^+^ cells (**C**) and mean fluorescence intensity (MFI) of CD69 expression (**D**) on MAIT cells were significantly higher in BALF than in peripheral blood mononuclear cells (PBMCs). *P < 0.05, **P < 0.01, ***P < 0.001.

To examine the involvement of MAIT cells in the lungs of sarcoidosis patients, we analyzed the proportion and expression of cell surface markers on MAIT cells in BALF (Fig. 6). The absolute number and proportion of MAIT cells were significantly higher in BALF from patients with parenchymal infiltration (pulmonary stage ≥ 2) than in those without parenchymal infiltration (pulmonary stage 0–1) (P = 0.002 and P = 0.02, respectively) (Fig. 6A,B). Furthermore, we compared CD69 expression levels on MAIT cells in peripheral blood with BALF from sarcoidosis patients. As shown in Fig. 6C,D, the proportion of CD69^+^ MAIT cells (Fig. 6C) and CD69 expressions on MAIT cells (Fig. 6D) were significantly higher in BALF than in peripheral blood (P = 0.0003 and P = 0.0005, respectively). Taken together, MAIT cells are especially infiltrated in inflammatory sites of the lungs and are highly activated, suggesting their involvement with the pathogenesis of sarcoidosis.

## Discussion

We found that MAIT cells were activated by *C. acnes* stimulation. In addition, the peripheral blood of sarcoidosis patients showed fewer MAIT cells than that of healthy controls. The peripheral blood of sarcoidosis patients also showed higher expression levels of CD69 and PD-1 on MAIT cells compared with healthy controls. CD69 expression levels were significantly correlated with ACE and sIL-2R levels, which are markers of clinical sarcoidosis disease activation. In addition, sarcoidosis patients with parenchymal infiltration showed significantly higher numbers and proportions of MAIT cells in BALF compared with sarcoidosis patients without parenchymal infiltration, indicating that MAIT cells notably infiltrated the inflammatory sites of sarcoidosis lungs and were highly activated. These results indicate a pathogenic role for MAIT cells in sarcoidosis. To our knowledge, this is the first study to identify an association between MAIT cells and sarcoidosis.

MAIT cells express an invariant TCR α chain paired with a limited set of Vβ chains (Vα7.2-Jα33 in humans and Vα19-Jα33 in mice) and are restricted by MR1^17,18^. MAIT cells are preferentially located in the gut lamina propria^17,19^, but are also found in the liver, lung, and peripheral blood^13,21–23^. Several studies have reported the involvement of MAIT cells in various types of diseases, including inflammatory diseases, metabolic diseases, infectious diseases, autoimmune diseases such as systemic lupus erythematosus (SLE), multiple sclerosis (MS), and inflammatory bowel disease (IBD), diabetes mellitus, and human immunodeficiency virus infection^24,38,40–49^. The activation marker CD69 on MAIT cells is more highly expressed in these inflammatory conditions than in controls. Similarly, we found that CD69 expression on MAIT cells was much higher in sarcoidosis patients than in healthy controls and was significantly correlated with the clinical biomarkers ACE and sIL-2R. ACE and sIL-2R are useful for evaluating sarcoidosis activity^34–37^. High baseline ACE levels have been reported to be correlated with improvements in lung function after treatment in sarcoidosis^37^. Therefore, the activity of MAIT cells might help decide therapeutic interventions such as corticosteroids or be used to assess the response of treatment.

We found that the proportion of MAIT cells in peripheral blood was lower in sarcoidosis patients than in healthy controls. Interestingly, decreased proportions of MAIT cells in peripheral blood have been reported in other systemic inflammatory diseases^13,38,41–47^. A possible reason for this finding is that MAIT cells migrate from the peripheral blood to inflamed tissues. MAIT cell accumulation is observed in organs involved in disease, such as the central nervous system of MS patients^41,49^, synovial fluid of rheumatoid arthritis patients^44^, and colons of IBD patients^42,43^. MAIT cells constitutively express IL-18Rα, and IL-18 upregulates cell surface expression of very late antigen 4 on MAIT cells, thereby inducing T cell migration^41^. Previous studies have reported that plasma IL-18 levels are negatively correlated with the numbers of MAIT cells in SLE and MS patients^38,41^ and are positively correlated with CD69 expression of MAIT cells in IBD and SLE patients^38,42^. Similarly, in the present study, serum IL-18 levels were significantly higher in sarcoidosis patients than in healthy controls, and we observed a significant correlation between the serum IL-18 concentration and CD69 expression level on MAIT cells in peripheral blood. Analysis of BALF in the current study revealed that MAIT cells had infiltrated the lungs of sarcoidosis patients, in particular those with parenchymal infiltration, and the activation marker of MAIT cells was significantly higher in BALF than in peripheral blood. These results suggest that MAIT cells migrate into the lungs through the IL-18/IL-18Rα pathway and are much more highly activated in inflamed sarcoidosis tissue. MAIT cells can produce considerable amounts of cytokines, such as IFN-γ and TNF-α, which are strongly associated with granuloma formation in sarcoidosis^2,3,6–8^. Thus, migration and activation of MAIT cells may contribute to the pathogenesis of sarcoidosis in the lungs through the production of cytokines such as IFN-γ and TNF-α.

Another possible reason for the lower proportion of MAIT cells in peripheral blood is the sustained activation and increased apoptosis of MAIT cells as indicated by their expression patterns of activated caspase, B cell lymphoma-2, CD25, and PD-1^38,43,47^. PD-1, TIM-3, and LAG-3 are co-inhibitory receptors that regulate excess T cell response and maintain immune balance. Sustained expression of these co-inhibitory receptors on T cells is considered to indicate an exhausted status in which their proliferative and cytotoxic abilities are lost in response to antigen stimulation^50–52^. Enhanced PD-1 expression on MAIT cells in patients with SLE has been shown to be associated with the low responsiveness of these cells^44^. In the present study, PD-1 was upregulated on sarcoidosis MAIT cells compared to healthy controls. However, there was no significant difference in TIM-3 and LAG-3 expression. Similarly, PD-1 on MAIT cells is expressed at a much higher level in patients with active tuberculosis compared to healthy controls; however, TIM-3 and LAG-3 are not^27^. Interestingly, PD-1 expression on MAIT cells was negatively correlated with the proportion of MAIT cells in peripheral blood. Thus, PD-1 might reflect the exhausted status of MAIT cells. Taken together, MAIT cells are activated in sarcoidosis patients, and sustained activation may be associated with a decrease in MAIT cells in peripheral blood.

Currently, two infectious agents are suspected to be related to sarcoidosis pathogenesis: *Mycobacterium spp*. and *Cutibacterium spp*^53–55^. Several studies have reported that MAIT cell proportions are lower in peripheral blood from tuberculosis patients^21,22,27^. Jiang *et al*. reported that MAIT cell levels are lower in peripheral blood from active tuberculosis patients and that MAIT cells from healthy controls react to mycobacterial antigen stimulation^27^. *C. acnes* is the only microorganism that has been isolated in bacterial cultures of sarcoidosis granulomas^28^. A previous immunohistochemical study using a monoclonal antibody against *C. acnes* reported a high frequency and specificity of *C. acnes* in sarcoid granulomas^29^. In addition, other investigations using quantitative polymerase chain reaction and *in situ* hybridization have shown that fragments of nucleic acids of *C. acnes* are present in sarcoid lymph nodes^30–32^. Our finding that MAIT cells can be activated by another suspected causative infectious agent supports the hypothesis that MAIT cells contribute to sarcoidosis pathogenesis.

In summary, the proportion of MAIT cells in peripheral blood was lower but more activated in patients with sarcoidosis than in healthy controls. In addition, MAIT cells notably infiltrated inflamed sites and were strongly activated in lungs of sarcoidosis patients. Therefore, MAIT cells are a potential target for sarcoidosis treatment. Inhibitory MR1 ligands or neutralizing antibodies are possible therapeutic agents for use in future clinical trials.

## Methods

### Patients

Forty sarcoidosis patients treated at Toho University Omori Medical Center and Shinjuku Tsurukame Clinic, and 28 age- and sex-matched healthy controls, were recruited. Diagnosis of sarcoidosis was based on histological findings of noncaseating epithelioid granulomas in addition to relevant clinical and radiological findings as specified by the American Thoracic Society/European Respiratory Society/World Association for Sarcoidosis and Other Granulomatous Disorders statement on sarcoidosis^56^. If a biopsy specimen was not available for histological examination, diagnosis was based on clinical and radiological consistency and exclusion of other diseases^56^. About 30% of patients were taking corticosteroids at the time of examination. ACE was measured by colorimetry^57^ and sIL-2R was measured by ELISA in a clinical laboratory (normal values: ACE, 8.3–21.4 U/L; sIL-2R, 145–519 U/mL). The stage of pulmonary lesions was evaluated by chest radiography as previously described^58^.

### Flow cytometric analysis

Peripheral venous blood samples were collected in heparin-containing tubes. PBMCs were purified by density-gradient centrifugation using BD vacutainer mononuclear cell preparation tubes with sodium heparin (Becton Dickinson and Company, NJ). BALF was collected with a fiber optic bronchoscope on the same day. Briefly, 50 mL of sterile 0.9% NaCl was administered three times to the right medial lobe or left lingular lobe. After each instillation, the saline was immediately withdrawn, and cells in BALF were purified in the same manner as PBMCs.

PBMC samples (2 × 10^6^/well) and BALF samples (2 × 10^6^/well) were incubated with Fc receptor-blocking reagent (BioLegend, San Diego, CA), and cell surface staining was performed with the following monoclonal antibodies and tetramers: anti-TCR pan-γ/δ-fluorescein isothiocyanate (FITC) (clone IM1571U) (Beckman Coulter, Indianapolis, IN), anti-CD69-Alexa Fluor 700 (clone FN50), anti-CD223 (LAG-3)-Alexa Fluor 700 (clone #874501), anti-mouse IgG1κ-Alexa Fluor 700 (clone MOPC-21), anti-CD3 allophycocyanin-H7 (clone SK7 [Leu-4]), anti-CD19-FITC (clone HIB19) (BD Biosciences, San Jose, CA), anti-CD366 (TIM-3)-phycoerythrin (clone F38-2E2) (R&D Systems, Minneapolis, MN), anti-CD279 (programmed death 1 [PD-1])-phycoerythrin (clone EH12.2H7), anti-mouse IgG1κ-phycoerythrin (clone MOPC-21), anti-CD161-peridinin chlorophyll/cyanine 5.5 (clone HP-3G10), anti-CD123-FITC (clone 6H6), anti-CD303 (blood dendritic cell antigen 2)-FITC (clone 201 A), anti-FcεR1α-FITC (clone AER-37), anti-CD1a-FITC (clone HI149), anti-CD34-FITC (clone 581), anti-CD11c FITC (clone 3.9), anti-CD14-FITC (clone HCD14) (BioLegend), anti–major histocompatibility complex 1 (MR1)/5-(2-oxopropylideneamino)-6-D-ribitylaminouracil tetramer-Brilliant Violet 421, and anti-CD1d/phosphate-buffered salts-57 tetramer-allophycocyanin (National Institutes of Health Tetramer Core Facility, Atlanta, GA). iNKT cells were defined as CD3^+^ Lin^−^ CD1d^+^ cells, and MAIT cells were identified as CD3^+^ Lin^−^ MR1^+^ CD161^high^ cells (Supplementary Fig. S1). An MR1 tetramer was generated to specifically detect MAIT cells^59,60^. To gate out cells that nonspecifically bound to tetramers, we used antibody cocktails against lineage markers. Lineage marker negative (Lin^−^) was defined as CD1a^−^, CD11c^−^, CD14^−^, CD19^−^, CD34^−^, CD123^−^, CD303^−^, TCR γ/δ^−^, and FcεR1α^−^ to exclude myeloid cells, B cells, and γδT cells. Data were acquired by fluorescence-activated cell sorting on an LSR FORTESSA analyzer (BD Biosciences), and the percentage of each cell population and mean fluorescence intensity (MFI) were analyzed with FlowJo software (FlowJo LLC, Ashland, OR).

### *In vitro* stimulation

PBMCs (1 × 10^6^/well) were isolated from the peripheral blood of healthy controls and suspended in 96-well U-bottom plates in RPMI 1640 medium (Thermo Fisher Scientific, Waltham, MA) supplemented with 10% fetal bovine serum and 2 mM l-glutamine (all from Thermo Fisher Scientific) without antibiotics. PBMCs were stimulated with *C. acnes* (1 × 10^8^/well) with anti-CD28 (clone CD28.2) (BioLegend). Cells were incubated for 12 h at 37 °C in a 5% CO_2_ incubator. After washing the cells, cell surface markers on MAIT cells were analyzed by flow cytometry.

### Cytokine measurement

Plasma from sarcoidosis patients and healthy controls was collected by density-gradient centrifugation of blood samples and frozen at −80 °C. Plasma cytokine levels were measured with a sandwich enzyme-linked immunosorbent assay for IL-18 (Medical & Biological Laboratories Co., Nagoya, Japan) in accordance with the manufacturer’s protocol.

### Statistical analysis

Statistical analysis was performed with the Fisher exact test, Wilcoxon signed rank test, and Mann-Whitney U test, as appropriate. Correlations between two groups were evaluated with Pearson’s correlation coefficient. A P value of less than 0.05 was considered to indicate statistical significance. All statistical analyses were done with GraphPad Prism version 7 (MDF Co., Ltd., San Diego, CA).

### Ethics statement and protocol approvals

The study was approved by the Ethics Committee of Toho University School of Medicine (protocol number A16112). All patients and healthy controls provided written informed consent that was approved by the institutional review board. All research was performed in accordance with these guidelines/regulations.

## Supplementary information

Supplementary materials

## Data Availability

The datasets analyzed during the current study are available from the corresponding author upon reasonable request. Researchers should obtain permission from the local ethics committee before making such a request.
