# Supplementary material for: Activation of mucosal-associated invariant T cells in the lungs of sarcoidosis patients
Source: Sci Rep. 2019 Sep 12;9:13181. doi: 10.1038/s41598-019-49903-6 (PMC6742655; doi:10.1038/s41598-019-49903-6)
Supplement: Supplementary file 1 — Supplementary materials [file 41598_2019_49903_MOESM1_ESM.pdf]

Supplementary Materials

Activation of mucosal-associated invariant T cells in the lungs of sarcoidosis patients

Hisayo Matsuyama, Takuma Isshiki, Asako Chiba, Tetsuo Yamaguchi, Goh Murayama, Yoshikiyo Akasaka, Yoshinobu Eishi, Susumu Sakamoto, Sakae Homma, Sachiko Miyake

Supplementary Table S1 Characteristics of healthy controls and sarcoidosis patients.

	HC (n= 7)	SA (n= 5)
Age (years)	33 ± 2	59 ± 11
Male, n (%)	6 (86)	3 (60)
Smoking history (never/ former/ current)	4/ 3/ 0	2/ 2/ 1
Duration of disease (years)	-	16 ± 9
Number of involved organs (1/ 2/ 3/ 4/ 5)	-	0/ 1/ 4/ 0/ 0
Stage of pulmonary lesion (0/ 1/ 2/ 3/ 4)	-	0/ 2/ 0/ 2/ 1
Ongoing treatment (none/ CS/ MTX)	-	0/ 1/ 1
Pathological proven, n (%)	-	5 (100)

n (%) or the mean ± standard deviation.

Abbreviations: HC Healthy control subjects, SA patients with sarcoidosis, CS corticosteroids, MTX methotrexate

Supplementary Figure S1

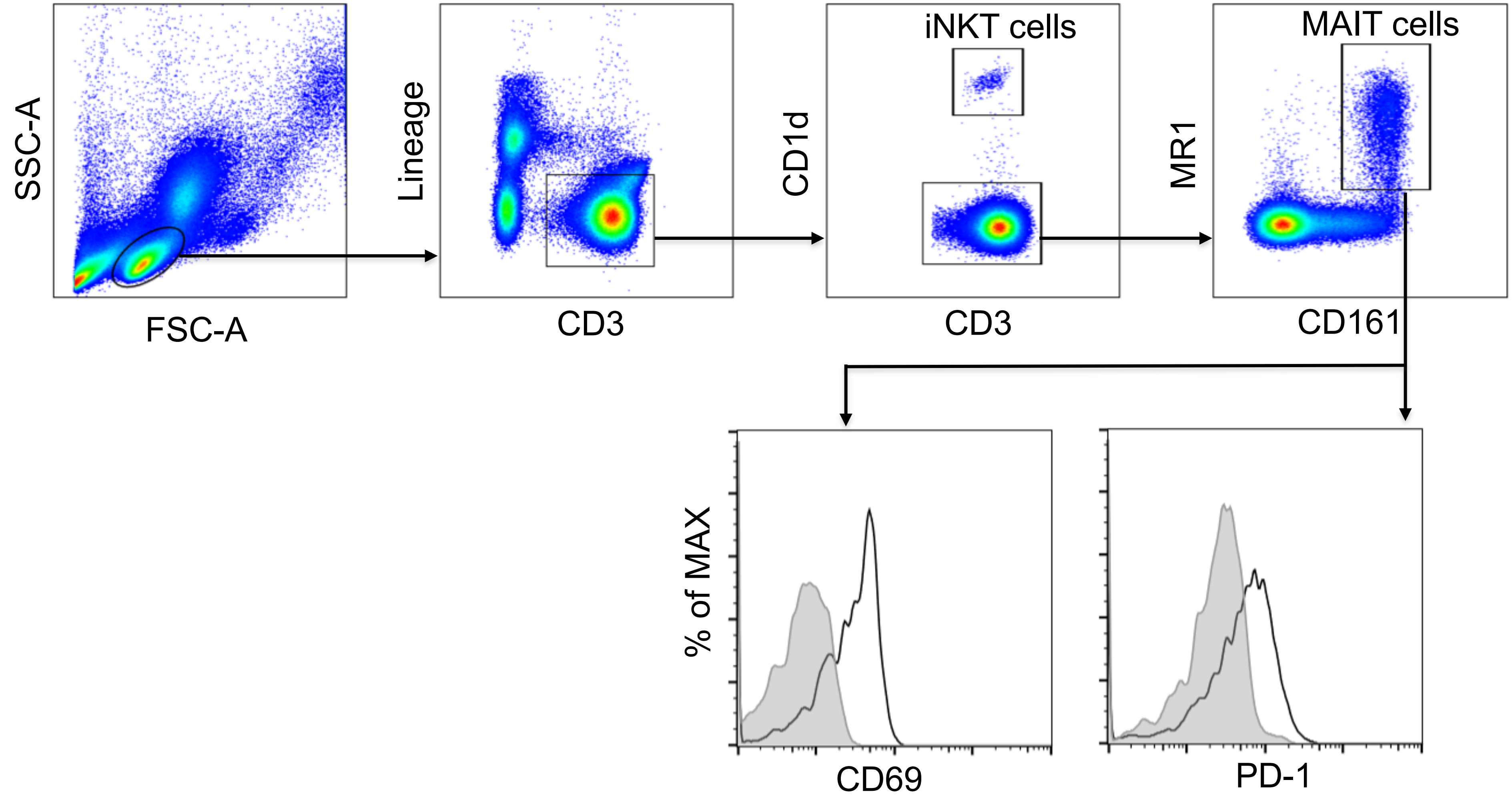

SUPPLEMENTARY FIGURE S1. Flow cytometry gating strategy for identifying invariant natural killer T (iNKT) cells and mucosal-associated invariant T (MAIT) cells in peripheral blood mononuclear cells and bronchoalveolar lavage fluid; representative flow plots and histograms are shown. After staining with monoclonal antibodies, the cells were analyzed with flow cytometry. Cells were gated on lymphocyte size by using forward scatter (FSC) and side scatter (SSC) and then gated on single cells, i.e., $CD3^{+}$ $Lineage^{-}$ $MR1^{+}$ $CD161^{high}$ cells. Lineage marker negative (Lin^{-}) was defined as $CD1a^{-}$, $CD11c^{-}$, $CD14^{-}$, $CD19^{-}$, $CD34^{-}$, $CD123^{-}$, $CD303^{-}$, T cell receptor γ/δ^{-}, and $Fc\varepsilon R1\alpha^{-}$. iNKT cells were defined as $CD3^{+}$ Lin^{-} $CD1d^{+}$ cells. MAIT cells were identified as $CD3^{+}$ Lin^{-} $MR1^{+}$ $CD161^{high}$ cells. CD69 and programmed death 1 (PD-1) expression of MAIT cells and percentages of $CD69^{+}$ and $PD\text{-}1^{+}$ MAIT cells are shown in histograms. The gray histograms are the isotype control.
